# Prosthodontic rehabilitation with all-on-four implant treatment combined CAD/CAM prosthesis in an oral cancer patient: a case report

**DOI:** 10.1186/s12903-024-04821-6

**Published:** 2024-09-13

**Authors:** Yi-Fang Huang, Chung-Ta Chang, Chih-Hung Lin, Yu-Fu Shen

**Affiliations:** 1 Department of General Dentistry, Chang Gung Memorial Hospital, No.5, Fuxing St, Guishan Township, Taoyuan County, 33305 Taiwan; 2 Graduate Institute of Dental and Craniofacial Science, College of Medicine, Chang Gung University, Taoyuan, 33302 Taiwan; 3 College of Medicine, Chang Gung University, Taoyuan, 33302 Taiwan; 4 School of Dentistry, College of Oral Medicine, Taipei Medical University, Taipei, 11031 Taiwan; 5 Department of Emergency Medicine, Far Eastern Memorial Hospital, New Taipei, 22056 Taiwan; 6 Graduate Institute of Medicine, Yuan Ze University, Taoyuan, 32003 Taiwan; 7 Center for Vascularized Composite Allotransplantation, Chang Gung Memorial Hospital, Taoyuan, 33305 Taiwan; 8 Department of Plastic and Reconstructive Surgery, Chang Gung Memorial Hospital, Taoyuan, 33305 Taiwan

**Keywords:** Microvascular free fibula flap, All-on-four implant therapy, CAD/CAM prosthesis, Oral cancer

## Abstract

**Background:**

The microvascular free fibula (MFF) flap is a reliable treatment modality for mandibular reconstruction and is suitable for dental implant placement after oncologic surgery. The most common issue with the MFF flap is its limited bone height, which typically results in excessive interarch space and complicates prosthodontic therapy. Overcoming the physical limitations from tumor excision and reducing the treatment time for prosthodontic rehabilitation to improve quality of life are critical clinical challenges.

**Case Presentation:**

A 64-year-old male with lower left gum and bilateral buccal cancer received a single-layer microvascular MFF flap to reconstruct a mandibular defect post-tumor excision. He underwent a bilateral modiolus Z-plasty combined with a skin flap debulking procedure to relieve oral contracture, achieving adequate mouth opening for prosthodontic rehabilitation. Scar tissue bands on the bilateral cheeks significantly affected retention and stability, hampering dental impression performance. The patient sought prosthodontic rehabilitation to enhance his chewing function and quality of life promptly. Prosthodontic rehabilitation with all-on-4 implant therapy, utilizing computer-aided design and computer-assisted manufacturing (CAD/CAM), was completed within one month.

**Conclusion:**

This case utilized the all-on-4 implant system to address the insufficient fibular height for conventional dental implant placements. Dental CAD/CAM was employed to mill custom prosthetic abutments and a large titanium framework for the implant bar overdenture, compensating for the excessive interarch space between the grafted fibula and maxilla. This treatment approach successfully shortened the prosthodontic rehabilitation time and overcame anatomical limitations.

## Background

The surgical removal of extensive oral malignancies involving multiple anatomical and functional subunits increases the difficulty of reconstruction. Extensive and complex defects of the head and neck after tumor excision inevitably result in the loss of oral functions, including chewing, swallowing, speech, and the senses of taste and smell [1]. Implant dentistry is the most effective prosthodontic treatment for restoring masticatory performance [2]. The microvascular free fibula (MFF) flap is a reliable treatment modality for mandibular reconstruction [3] and is suitable for dental implant placement after oncologic surgery [4]. The most commonly encountered problem with the MFF flap [5] is its limited bone height, which usually causes excessive interarch space and complicates prosthodontic therapy [6].

Many treatment modalities, such as distraction osteogenesis and the double-barrel fibula technique of MFF reconstruction, are often utilized to increase bone height and shorten the vertical distance to the occlusal plane [7]. However, distraction osteogenesis requires a significant amount of time for bone growth, delaying oral function recovery. Limited number of studies have revealed positive results from placing dental implants in a vascularized double-barrel fibula bone to achieve functional mandibular reconstruction [8]. Overcoming the physical limitations derived from tumor excision and shortening the treatment time of prosthodontic rehabilitation to recover quality of life are critical clinical issues.

An oral cancer patient received a single-layer MFF flap for mandibular reconstruction after tumor excision. The all-on-four implant treatment, combined with dental computer-aided design and computer-assisted manufacturing (CAD/CAM), was used to produce a prosthesis for oral rehabilitation. This treatment modality successfully restored chewing function in a short time.

### Case presentation

**Fig. 1:**
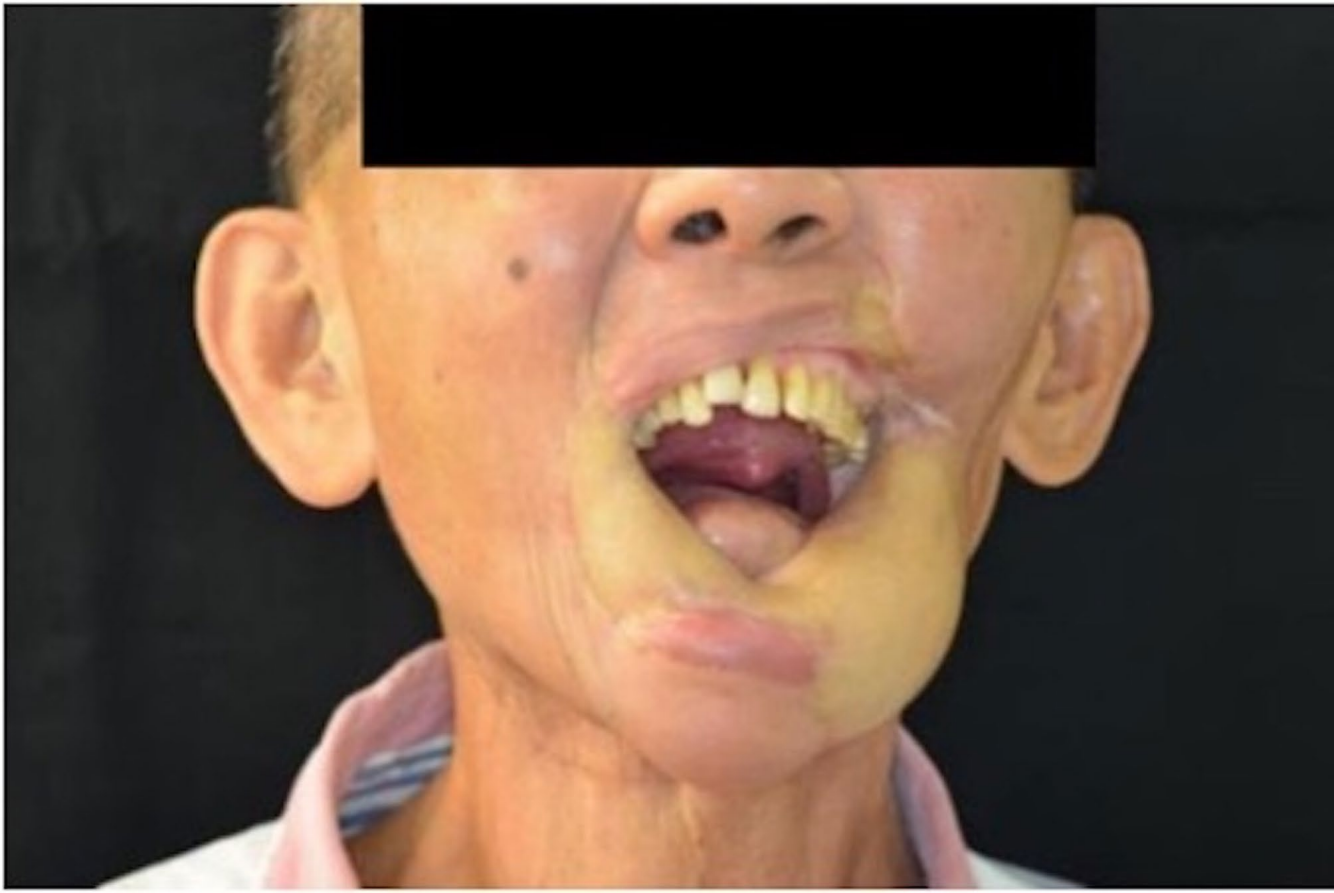
The mouth opening of the patient was adequate for prosthodontic rehabilitation after a bilateral modiolus Z-plasty combined skin flap debulking procedure to relief the oral contracture

**Fig. 2:**
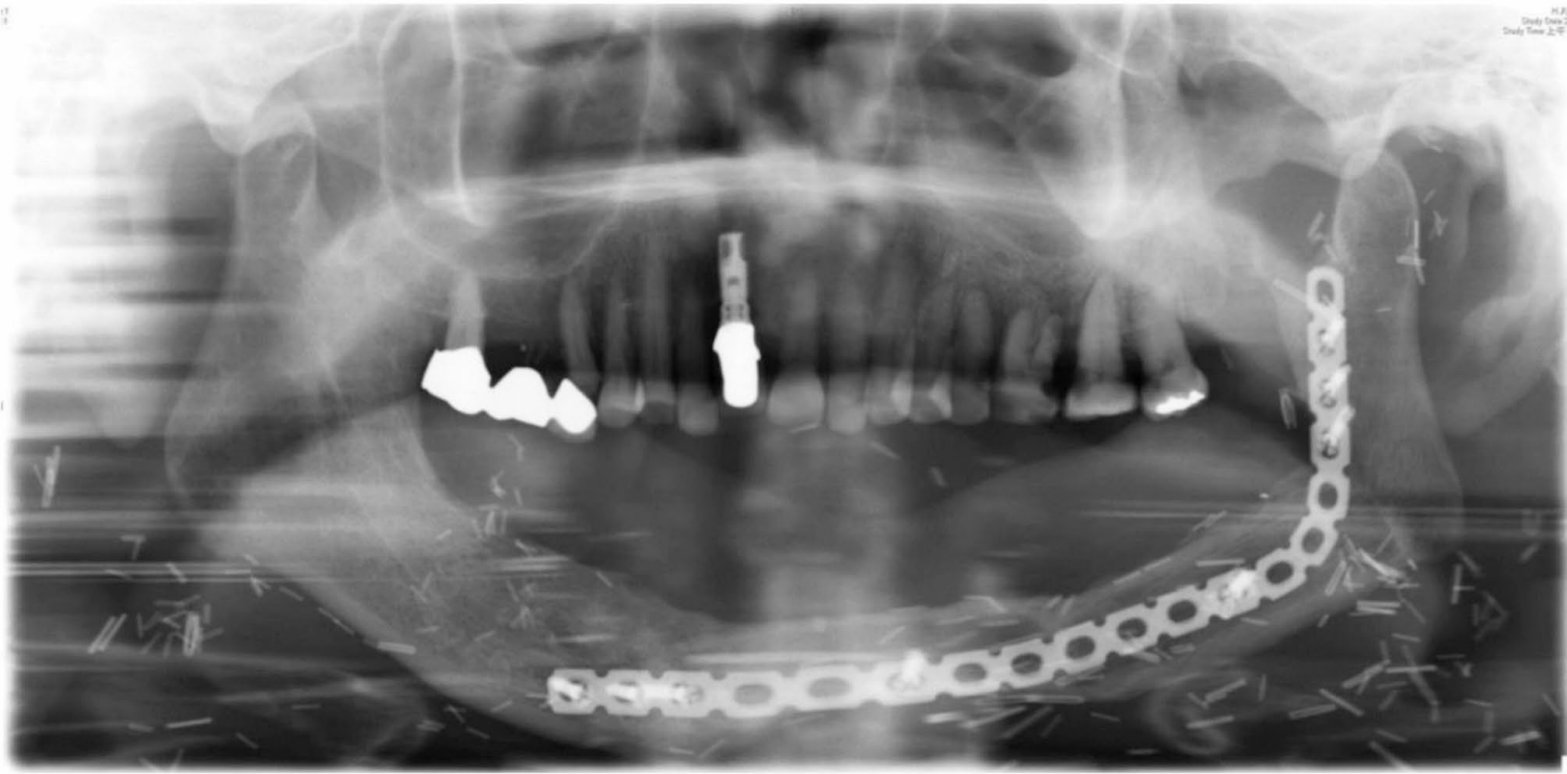
The patient encountered the problem that the MFF flap is the limited bone height, which usually causes excessive interarch space and increases the difficulty of prosthodontic therapy

**Fig. 3:**
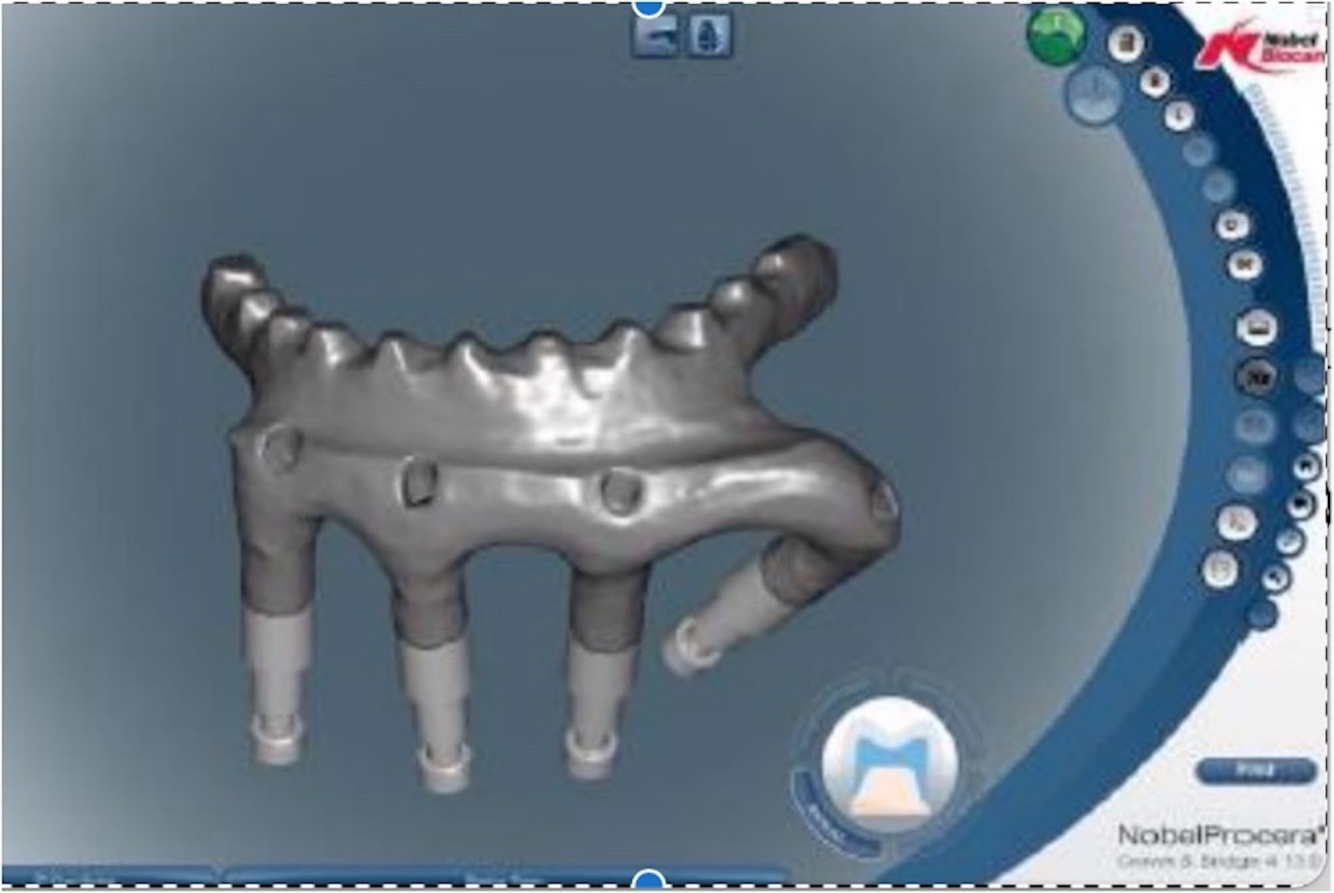
The CAD/CAM system was used to manufacture the massive framework of implant bar overdenture derived from the excessive space between the fibula reconstructed mandible and antagonist maxillary teeth

**Fig. 4:**
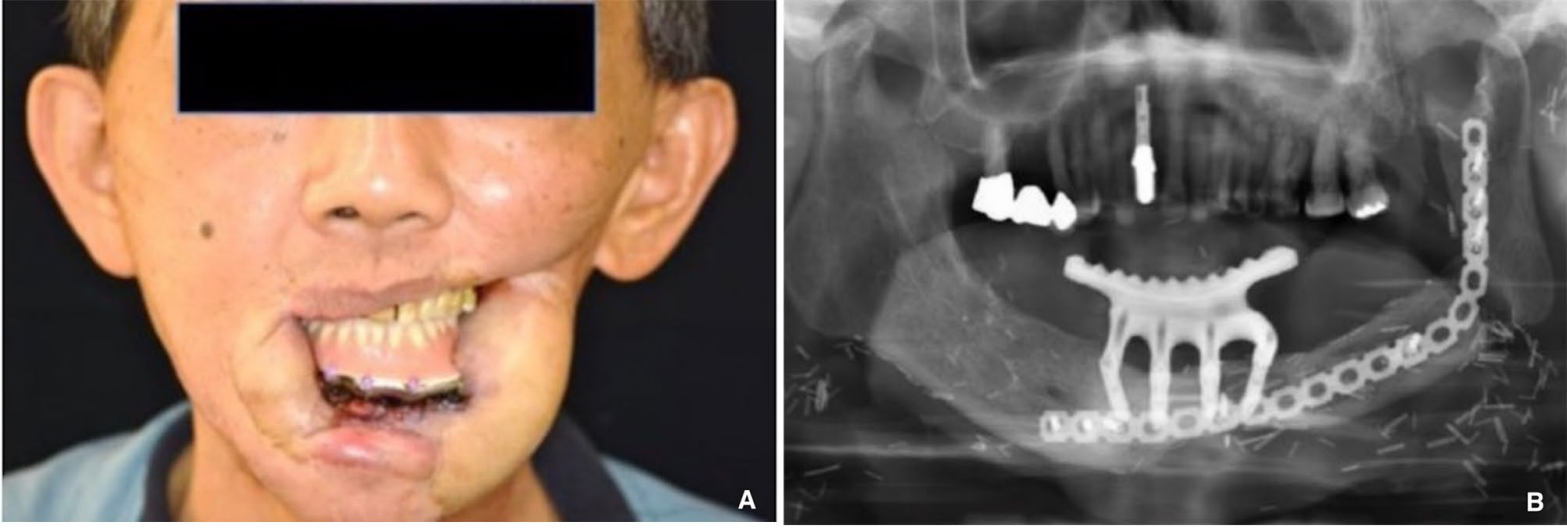
All-on-four implant therapy combined with CAD/CAM prosthesis successfully overcame the limitation of insufficient grafted fibular height and shortened the treatment tome of prosthodontic rehabilitation. **A**. Extra-oral view with implant bar overdenture. **B**. panoramic view with implant bar overdenture

A 64-year-old male with lower left gum and bilateral buccal cancer (cT2N0, pT2N0M0) with oral commissure defects underwent an MFF flap to reconstruct the mandibulectomy defect after tumor excision. Ankylosis and malposition of the left condyle restricted mandibular lateral movement post-oncologic and reconstructive surgery. The patient spent two years undergoing tumor excision, reconstructive surgery, and radiotherapy. After oncologic and reconstructive surgery, the intraoral wound healing was stable, but the patient suffered from a liquid diet and limited mouth opening. He was strongly motivated to receive prosthodontic rehabilitation to improve his chewing function and quality of life once his oncologic condition was controlled. Following a bilateral modiolus Z-plasty combined with a skin flap debulking procedure to relieve the oral contracture, the patient’s mouth opening (a 33 mm interincisal distance) was adequate for prosthodontic rehabilitation (Fig. 1). An implant prosthesis was recommended to restore the edentulous mandible due to lack of vestibular depth and bilateral scar tension, which would affect the retention and stability of a removable denture. As the patient desired to recover masticatory function promptly and strongly rejected any plastic surgery for dental implant placement, the all-on-four implant (NobelBiocare^®^) therapy was considered to overcome the insufficient bone height of the MFF flap (Fig. 2). An intraoral scan replaced the conventional dental impression to transfer the implant fixture for prosthetic fabrication seven days after implant placement. A CAD/CAM system was used to produce the large volume derived from the excessive space between the fibula-reconstructed mandible and antagonist maxillary teeth (Fig. 3). The implant bar overdenture was delivered with low insertion torque, which was torqued to 20 Ncm three months later. The prosthodontic rehabilitation, from implant placement to finished prosthetic reconstruction, took only one month. The patient has maintained regular annual dental follow-ups for the last seven years, with no complications such as screw fractures, loosening, or superstructure fractures of the all-on-four implant therapy. Post-prosthetic rehabilitation, the patient had a normal diet, and both masticatory function and quality of life were greatly improved (Fig. 4). The case received regular dental follow-ups every six months, with no implant or prosthodontic complications.

## Discussion

The all-on-four concept is well-established for the edentulous mandible and maxilla with insufficient bone volume. Clinically, this technique has favorable survival rates for the immediate rehabilitation of fully edentulous jaws using two posterior tilted and two axial implants. The increased implant length of the tilted implant can enhance the contact surface between the implant fixture and bone, promoting osteointegration and successfully overcoming anatomical limitations to replace bone graft surgery [9, 10]. Most oral cancer patients reject bone grafts for implant therapy due to unpleasant experiences with oncologic surgery in the past. The all-on-four implant therapy is a good treatment modality to recover chewing function quickly and avoid a fibula graft for increasing bone height.

In this case, the post-surgical scar tissue band on the bilateral cheeks restricted dental impression for implant fixture transfer. An intraoral scan successfully overcame this anatomical limitation. CAD/CAM offers a novel solution to produce 3D-printed osteotomy guides and reconstructive plates for bony defect reconstruction. Previous studies indicated that fabricating custom prosthetics is an important aim for developing innovative techniques in the future [11]. Prosthodontic-driven treatment planning for dental implant placement is challenging for oral cancer patients due to the vast anatomical alterations post-tumor excision [2]. Bone position and volume limit implant placement, necessitating custom abutments and prosthetics to compensate for the bone-driven implant treatment outcome. This case utilized CAD/CAM to produce not only the custom prosthodontic abutment but also the framework for the implant bar overdenture. Due to the limited width of the fibula, a customized abutment was used to overcome the large interarch space between the MFF flap and antagonist dentition. The customized abutment was fabricated from a CAM system to mill the titanium block, with the screw made by the NobelBiocare^®^ original factory. This pioneering technique successfully overcame the massive volume metal framework derived from the excessive space between the MFF flap and antagonist occlusal plane. Conventional investment and casting techniques cannot produce giant metal frameworks because porosities impact the structural strength. The patient continuously received regular dental follow-up every six months over three years, with no prosthodontic or implant complications to date. In this case, the milling of a titanium block by a CAD/CAM system successfully solved this difficult problem that could not be handled in the past, effectively overcoming the limitations of physiological conditions.

## Conclusions

This case successfully employed the All-on-4 implant system to address the challenge posed by insufficient fibular height, which typically complicates conventional dental implant placements. The innovative approach capitalized on dental CAD/CAM technology to meticulously mill a custom prosthetic abutment, alongside an extensive titanium framework for the implant bar overdenture. This framework was precisely designed to accommodate the excessive interarch space between the grafted fibula and the maxilla, a common complication in such reconstructive scenarios. The All-on-4 system, renowned for its efficiency and effectiveness in full-arch rehabilitation, was pivotal in overcoming the anatomical limitations presented by the patient’s condition. By leveraging advanced digital dentistry tools, the treatment not only enhanced the precision and fit of the prosthetic components but also significantly expedited the prosthodontic rehabilitation process. This method circumvented the need for more invasive and time-consuming traditional procedures, offering a streamlined solution that catered to the patient’s unique anatomical constraints. The synergy of the All-on-4 system and cutting-edge CAD/CAM technology thus played a crucial role in achieving a successful outcome, ensuring the stability and functionality of the dental prosthesis while also optimizing the overall treatment timeline. This case exemplifies the potential of modern dental techniques in addressing complex reconstructive challenges, ultimately improving patient care and outcomes through innovative solutions.

## Data Availability

The data sets generated and/or analyzed during the present study are available from the corresponding author on reasonable request.

## Abbreviations

CAD/CAM: Computer aided design and computer assisted manufacturing
MFF: Microvascular free fibula
